# Fibronectin and Cyclic Strain Improve Cardiac Progenitor Cell Regenerative Potential* In Vitro*

**DOI:** 10.1155/2016/8364382

**Published:** 2016-08-16

**Authors:** Kristin M. French, Joshua T. Maxwell, Srishti Bhutani, Shohini Ghosh-Choudhary, Marcos J. Fierro, Todd D. Johnson, Karen L. Christman, W. Robert Taylor, Michael E. Davis

**Affiliations:** ^1^Wallace H. Coulter Department of Biomedical Engineering, Emory University School of Medicine, Atlanta, GA 30322, USA; ^2^Division of Cardiology, Department of Medicine, Emory University School of Medicine, Atlanta, GA 30322, USA; ^3^Department of Bioengineering, Sanford Consortium for Regenerative Medicine, University of California, La Jolla, San Diego, CA 92037, USA; ^4^Children's Heart Research and Outcomes Center, Emory University and Children's Healthcare of Atlanta, Atlanta, GA 30322, USA

## Abstract

Cardiac progenitor cells (CPCs) have rapidly advanced to clinical trials, yet little is known regarding their interaction with the microenvironment. Signaling cues present in the microenvironment change with development and disease. This work aims to assess the influence of two distinct signaling moieties on CPCs: cyclic biaxial strain and extracellular matrix. We evaluate four endpoints for improving CPC therapy: paracrine signaling, proliferation, connexin43 expression, and alignment. Vascular endothelial growth factor A (about 900 pg/mL) was secreted by CPCs cultured on fibronectin and collagen I. The application of mechanical strain increased vascular endothelial growth factor A secretion 2–4-fold for CPCs cultured on poly-L-lysine, laminin, or a naturally derived cardiac extracellular matrix. CPC proliferation was at least 25% higher on fibronectin than that on other matrices, especially for lower strain magnitudes. At 5% strain, connexin43 expression was highest on fibronectin. With increasing strain magnitude, connexin43 expression decreased by as much as 60% in CPCs cultured on collagen I and a naturally derived cardiac extracellular matrix. Cyclic mechanical strain induced the strongest CPC alignment when cultured on fibronectin or collagen I. This study demonstrates that culturing CPCs on fibronectin with 5% strain magnitude is optimal for their vascular endothelial growth factor A secretion, proliferation, connexin43 expression, and alignment.

## 1. Introduction

The field of cardiac progenitor cells is complex and controversial. Much attention recently has been devoted to determining if c-kit positive cardiac progenitor cells (CPCs) contribute to endogenous myocardial regeneration [1–5]. While this is an important question, it does not address the full regenerative potential of CPCs. In fact, regeneration of myocardial tissue is improved by exogenous cell implantation [6–8]. CPCs as cell therapy in ischemic heart disease have a measurable cardiac benefit in the clinic [9]. The CPC regenerative potential can be improved* in vivo* by preconditioning CPCs before delivery or by delivering CPCs with an engineered vehicle [9, 10]. Engineered microenvironments can improve the CPC regenerative potential* in vitro* for applications such as lab-on-a-chip. It is of great interest to elucidate CPC-microenvironment interactions for the successful design of therapeutics.

The myocardium is a complex and dynamic tissue. Two signaling cues present in the myocardium are extracellular matrix and cyclic mechanical strain. It is unknown if cyclic strain affects CPC behavior or if these potential effects are dependent on the underlying substrate. All major cardiac cell types elongate and align perpendicular to an applied strain* in vitro* [11]. Further, mechanical strain regulates cell proliferation, hypertrophy, differentiation, and paracrine signaling [12, 13]. Extracellular matrix components alter CPC proliferation and differentiation [14]. To test the effect of extracellular matrix protein and mechanical strain on CPC behavior, a simplified, biomimetic approach based on endogenous signaling cues was taken.

Healthy myocardium is composed of collagen I, collagen III, fibronectin, laminin, collagen IV, elastin, and proteoglycans [15, 16]. Endogenous CPCs exist in niches composed of laminin and fibronectin in low strain regions of the myocardium [17]. During development and disease, the myocardial extracellular matrix and mechanical strain are altered. Myocardial infarction serves as an example herein as these structural changes are well characterized and CPC therapy is currently being evaluated in these patients [18]. After a myocardial infarction, there is an initial decrease in collagen content and organization followed by increased fibronectin and collagen deposition [15, 19–23]. Global strains of 18% in the healthy myocardium decrease to 7% following myocardial infarction [24, 25]. To systematically evaluate the influence of mechanical strain and extracellular matrix components on CPC growth factor secretion, proliferation, connexin43 expression, and alignment, this study uses culture conditions spanning a controlled range of signaling cues.

## 2. Materials and Methods

### 2.1. Cardiac Progenitor Cell Isolation and Characterization

All animal work was approved by Emory University's Institutional Animal Care and Use Committee. CPCs were isolated from adult male Sprague-Dawley rats (about 250 g) by removing the heart and homogenizing the tissue. The tissue homogenate was further digested with 1 mg/mL type-2 collagenase Hank's balanced salt solution (Worthington Biochemical) and passed through a 70 *μ*m filter. Dynabeads (Dynal) were conjugated to a c-kit antibody (Santa Cruz H-300). Cells were then incubated with beads for 2 hours at 37°C prior to magnetic sorting. Sorted cells were plated on a T-75 tissue culture flask and expanded to confluence in growth media (Ham's F-12 (Mediatech) + 10% fetal bovine serum (Atlanta Biologicals) + 0.1 *μ*g/mL basic fibroblast growth factor (Sigma) + 10 ng/mL leukemia inhibitory factor and human recombinant (Sigma) + 1x penicillin-streptomycin-glutamine (Cellgro)). After clonal expansion, CPCs were characterized by flow cytometry of c-kit (Santa Cruz H-300). Only clones with >90% c-kit expression were used for subsequent studies.

### 2.2. Cardiac Extracellular Matrix Isolation and Characterization

Decellularized porcine ventricular extracellular matrix was obtained and processed as previously described [26–28]. Briefly, porcine ventricular tissue was isolated and cut into small rectangular pieces, rinsed in phosphate buffered saline (PBS, Fisher), and decellularized using 1% sodium dodecyl sulfate (SDS, Fisher) for 4-5 days. The decellularized cECM was then rinsed with water overnight, frozen at −80°C overnight, lyophilized (Labconco) overnight, and milled into a fine powder. The powder was digested using pepsin at 1 mg/mL in 0.1 M HCl (Fisher) for two days, as modified from a previously published protocol, at a ratio of 10 : 1 of ECM matrix to pepsin [28, 29]. The material was then raised to a basic pH by adding 1 M NaOH (Fisher) and brought to a salt concentration of 1x PBS through the addition of 10x PBS. Then, the material was brought to physiological pH of 7.4 using HCl and NaOH and diluted to 2 mg/mL using 1x PBS. The cECM was then frozen at −80°C overnight, lyophilized for 24 hours (Labconco), and stored at −80°C prior to use. Matrix solutions were made by reconstituting cECM in sterile water and then diluted to 1 mg/mL in 100 mM acetic acid.

### 2.3. Silanization of Bioflex Plates

Working in a fume hood, 2 mL of 1.0 M NaOH (Sigma) was added to each well of a 6-well Bioflex plate (Flexcell International) and incubated for 1 hour at room temperature. Plates were washed in ddH_2_O thrice for five minutes. With the lids on, each well was treated with 1 mL of 4% (v/v) aminopropyltriethoxysilane (APTES; Sigma) in acetone for 10 minutes at room temperature. Plates were washed three times, treated with 2 mL 0.5% glutaraldehyde (Sigma) in ddH_2_O at room temperature for 30 minutes, and washed again. Matrix proteins were diluted to 100 *μ*g/mL, with 1 mL for each well, as follows: naturally derived cardiac extracellular matrix (cECM; porcine) in 100 mM acetic acid, collagen I (COL; rat tail, Invitrogen) in 100 mM acetic acid, fibronectin (FN; human, BD Bioscience) in 1x PBS, laminin (LN; mouse, BD Bioscience) in 1x PBS, and poly-L-lysine (Sigma) in ddH_2_O. Matrix proteins were added to the appropriate wells and plates were incubated for 1 hour at 37°C. Plates were then washed once for 5 minutes in 1x PBS and treated with 1 mL of 1 M ethanolamine (Sigma) in ddH_2_O (pH 7.0) for 20 min to quench unreacted glutaraldehyde. Plates were washed three times with 1x PBS. To sterilize, plates were kept under ultraviolet light for 1 hour prior to use.

### 2.4. Application of Mechanical Tension

CPCs were seeded at a density of 4 × 10^5^ cells/well on functionalized Bioflex plates and incubated in treatment media (Ham's F-12 (Mediatech) + 0.1 *μ*g/mL basic fibroblast growth factor (Sigma) + 1x insulin transferrin selenium (Cellgro) + 1x penicillin-streptomycin-glutamine (Cellgro)) for 6 hours prior to the application of mechanical tension. Tensile strain was applied through a Flexcell 5000 (Flexcell International). For this, Bioflex plates were loaded onto 25 mm cylindrical loading posts. A cyclic sinusoidal strain regimen of 1 Hz and 0.5 duty cycle, with an elongation magnitude of 5, 10, or 15%, was applied to the plates for 24 hours. Cells were maintained in 5% CO_2_ at 37°C throughout.

### 2.5. Strain Transfer Video Microscopy

Untreated StageFlexer membranes (Flexcell International) were functionalized as described above. For improved visualization, CPCs were incubated for 2 hours at 37°C with anti-c-kit antibody conjugated Dynabeads prior to being seeded at 5 × 10^5^ cells/membrane. Following 6-hour incubation in growth media, membranes were placed in a StageFlexer (Flexcell International) attached to the Flexcell 5000. A cyclic sinusoidal strain regimen of 1 Hz and 0.5 duty cycle, with elongation magnitudes of 5, 10, or 15%, was applied and bright field video was captured on an upright microscope (Amscope) with ToupView 3.7 at 5–20 frames per second. To quantify, a single cell containing two or more beads was identified. The distance between the two beads was traced in FIJI and strain was computed. This “measured strain” is compared to the strain reported by the Flexcell 5000.

### 2.6. Growth Factor ELISAs

After 24 hours of mechanical strain, conditioned media were immediately collected from the wells and stored at −20°C. ELISA kits were purchased from RayBiotech for stem cell factor, hepatocyte growth factor, platelet-derived growth factor, and vascular endothelial growth factor A. The insulin-like growth factor 1 ELISA was purchased from Thermo Fisher Scientific. Experiments were performed according to the manufacturer's protocol. Briefly, diluted conditioned media or standard was added to the appropriate well and incubated for 2.5 hours at room temperature. Following washing, the appropriate biotin-conjugated antibody was added to each well and incubated for 1 hour at room temperature. Following washing, a horseradish peroxidase-conjugated streptavidin solution was added to each well and incubated for 45 minutes at room temperature. After additional washing, substrate was added to each well and incubated for 30 minutes at room temperature. Finally, stop solution was added to each well. Plates were read immediately at 450 nm on a BioTek Synergy2 spectrophotometer.

### 2.7. Western Blot

Cells were lysed in NP-40 lysis buffer with protease and phosphatase inhibitors and incubated overnight at 4°C. Lysates were spun for 5 minutes at 10,000 ×g and the supernatant was collected to remove insoluble protein. Protein quantification was performed by microBCA (Thermo Scientific) according to the manufacturer's protocol. Samples were prepared by adding 35 *μ*g protein to appropriate amounts of 5x Laemmli buffer and water to yield a final volume of 35 *μ*L and then boiled for 8 minutes at 95°C. Each sample was then loaded on 10% or 12% SDS-PAGE gel. Electrophoresis was performed and gels were transferred to nitrocellulose membranes. Membranes were immediately blocked with 3% milk in Tris-buffered saline with 1% Tween-20 (TBS-T) for 1 hour at room temperature. Membranes were washed 3 times in 1x TBS-T and then immersed in a 1: 1000 primary antibody (PCNA: Santa Cruz; Cnx43: Sigma; GAPDH: Santa Cruz). All antibody solutions were made in 1% milk in 1x TBS-T and incubated with membranes overnight at 4°C prior to 3 washes with 1x TBS-T. Membranes were incubated at room temperature for 1 hour in 1: 5000 dilution of secondary antibody. The secondary antibody was HRP-conjugated goat anti-rabbit or goat anti-mouse (Bio-Rad). Membranes were exposed on film and results were quantified with ImageJ.

### 2.8. Fluorescence Microscopy

After 24 hours of mechanical strain, cells were immediately fixed in 4% paraformaldehyde (Sigma) for 20 minutes at room temperature. Following three washes in 1x PBS, the cells were permeabilized with 0.1% triton in (Sigma) 1x PBS. Cells were again washed and then blocked in 3% bovine serum albumin (Sigma) for 1 hour at room temperature. Cells were then stained with 10 *μ*g/mL fluoresceinyl-maleimide (Sigma) for 1 hour at room temperature in the absence of light, followed by washing, and then 1 *μ*g/mL DAPI (Invitrogen) for 10 minutes. Select wells were additionally stained for aurora B (Abcam). Cells were washed prior to imaging on an Olympus IX70 inverted fluorescent microscope.

Cell characteristics were quantified by importing images into CellProfiler 2.1.0. The images were analyzed according to the following pipeline: (1) images were resized by 0.25; (2) images were converted to greyscale; (3) nuclei were identified by intensity using a global threshold, automatic smoothing, and a threshold correction factor of 1; (4) cells were identified around nuclei by intensity through propagation with per object Otsu thresholding, automatic smoothing, and a threshold correction factor of 0.8; (5) cell size, shape, and orientation were measured; (6) images were exported to spreadsheet. Exported data was compiled to calculate cell size, spread area, and alignment. Alignment scores were calculated by measuring the angle between the major axis of each cell and the horizon, taking the standard deviation of all the angles and computing 100 *∗* the percent difference of the standard deviation from a Gaussian distribution or 100*∗*(90/sqrt(3) − stdev)/(90/sqrt(3)). Circular math was employed to correct for the orientation of cells in orthogonal images.

### 2.9. Statistics

All data was analyzed by one-way ANOVAs to establish specific effects of matrix or strain except where noted. All one-way ANOVA posttests are Tukey's multiple comparison tests allowing for the comparison of all groups to each other. GraphPad Prism 5 was used for all statistical analysis.

## 3. Results and Discussion

### 3.1. CPCs Strain with Underlying Membrane

**Figure 1:**
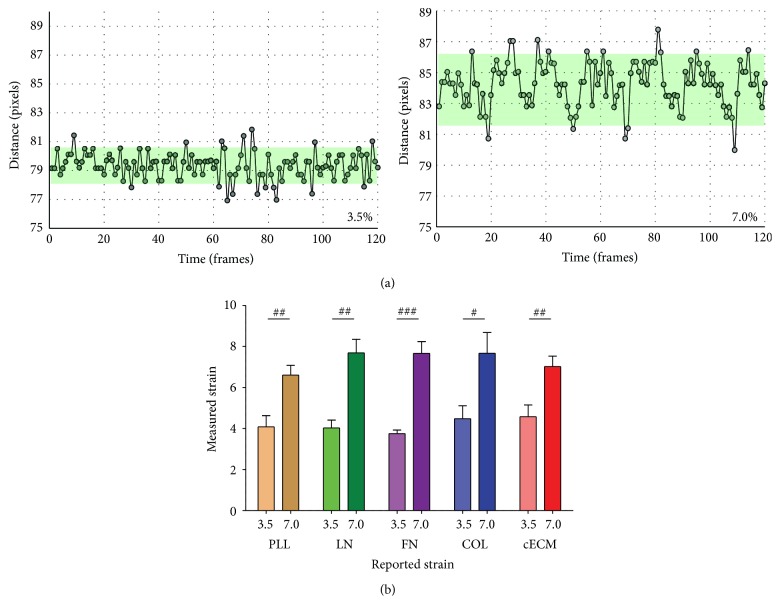
Cell strain. CPCs were seeded on the appropriate matrix for 6 hours. Video microscopy (19 frames per second) captured the motion of beads tethered to CPCs under cyclic 1 Hz strain. (a) Representative distance tracings from CPCs cultured on cECM; programmed strain is reported on graph. (b) Strain magnitude effects on measured strain. One-way ANOVA with Tukey's multiple comparison test; bars represent mean + SEM; ^#^ *p* < 0.05, ^##^ *p* < 0.01, and ^####^ *p* < 0.001; *n* = 3–10; 3.5, 7.0: strain magnitude (%), PLL (brown): poly-L-lysine, LN (green): laminin, FN (purple): fibronectin, COL (blue): collagen I, and cECM (red): naturally derived cardiac extracellular matrix.

To determine if CPCs cultured under the same strain magnitude experienced similar strains, independent of extracellular matrix coating, live cell video microscopy was performed using a StageFlexer. A notable (*p* < 0.05) increase in cell strain was observed with an increase in applied strain (Figure 1). The measured strain corresponded with the strain reportedly generated by the StageFlexer. This device has a smaller volume than the Bioflex 6-well plate used in the remainder of the study, limiting the maximum achievable strain magnitude to 7%. No differences in measured strain were present between matrix groups.

This suggests that regardless of the extracellular matrix coating CPCs grossly received the same applied strain. Any differences observed between matrix coating groups of the same strain magnitude will therefore be due to biochemical input from the extracellular matrix. Two other points are important for interpreting the data. First, the naturally derived cardiac extracellular matrix (cECM) contains each of the other matrix proteins evaluated in this study and represents a complex mixture. Second, the stiffness of PDMS (Bioflex plates) is an order of magnitude lower than tissue culture polystyrene and therefore even “static” conditions may not represent typical culture conditions. The effect of stiffness on CPC behavior is evaluated elsewhere [30, 31].

### 3.2. Strain Induces Vascular Endothelial Growth Factor Secretion

The CPC secretome may confer regenerative potential through growth factors [9]. Growth factors aid in recruitment or differentiation of endogenous progenitor cells and the survival of mature endogenous cells. Toward this end, several growth factors were examined in the conditioned media of strained CPCs. For all of the following studies, four matrix proteins (laminin: LN, fibronectin: FN, collagen I: COL, and cECM) and poly-L-lysine were evaluated at four strain magnitudes of 0, 5, 10, and 15%.

**Figure 2:**
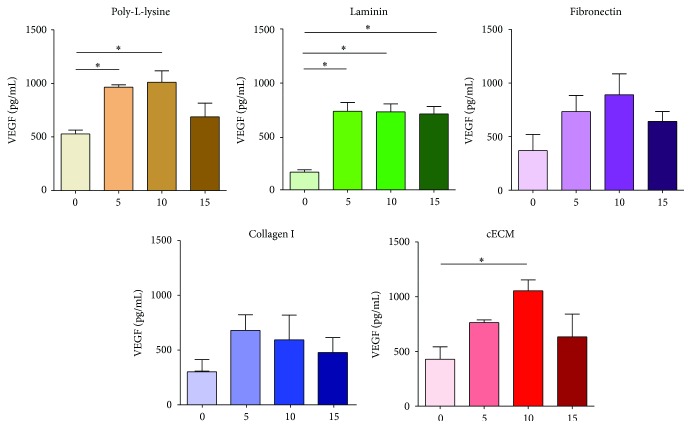
Strain improves VEGF secretion in CPC conditioned media. CPCs were seeded on each matrix for 6 hours and then cyclic strain was applied for 24 hours. Conditioned media were evaluated by ELISA. One-way ANOVA with Tukey's multiple comparison test; bars represent mean + SEM; ^*∗*^ *p* < 0.05; *n* = 3-4; *x*-axis: 0–15: strain magnitude (%) and VEGF: vascular endothelial growth factor A.

Vascular endothelial growth factor A (VEGF) was detected in CPC conditioned media. There was a marked increase in VEGF (Figure 2) in conditioned media from CPCs cultured on LN in the presence of 5, 10, and 15% (730, 729, and 699 pg/mL, resp.) strain as compared to unstrained groups (159 pg/mL; *p* < 0.01). Similarly, conditioned media from CPCs strained at 10% (1048 pg/mL) on cECM also showed higher VEGF than in unstrained controls (420 pg/mL; *p* < 0.05). FN and COL induced VEGF secretion at all strain magnitudes. This suggests that strain is an important modulator of VEGF secretion by CPCs. Extracellular matrix did not change VEGF concentration at a given strain magnitude. This data suggests that static culture of CPCs is detrimental to VEGF secretion. In addition to its angiogenic effects, VEGF acts through paracrine and autocrine effects to increase Cnx43 expression in myocytes [32, 33]. Neither platelet-derived growth factor nor insulin-like growth factor or stem cell factor was detected. Hepatocyte growth factor was detected in CPC conditioned media in low quantities (<10 *μ*g/mL, data not shown). Hepatocyte growth factor plays a role in cell proliferation, migration, survival, and angiogenesis [34]. While strain magnitude affected hepatocyte growth factor concentration, given the low absolute concentrations, these effects may not be biologically relevant.

### 3.3. CPC Proliferation Increased on Fibronectin

**Figure 3:**
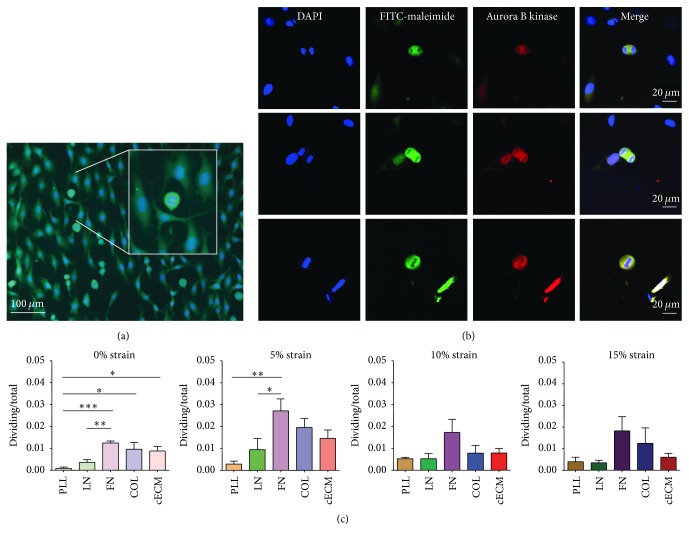
Fibronectin increases CPC division at low strain magnitudes. CPCs were seeded on each matrix for 6 hours and then cyclic strain was applied for 24 hours. (a) Representative image showing nuclear condensation and cytokinesis. Blue: DAPI and green: FITC-maleimide. Insert shows magnification of a dividing cell. (b) Representative image of aurora B kinase staining. Blue: DAPI, green: FITC-maleimide, and red: aurora B kinase. (c) Quantified results; one-way ANOVA with Tukey's multiple comparison test; bars represent mean + SEM; ^*∗*^ *p* < 0.05, ^*∗∗*^ *p* < 0.01, and ^*∗∗∗*^ *p* < 0.001; *n* = 4–6; PLL (brown): poly-L-lysine, LN (green): laminin, FN (purple): fibronectin, COL (blue): collagen I, and cECM (red): naturally derived cardiac extracellular matrix.

An increase in CPC number via proliferation would improve their regenerative potential by increasing the number of cells available for therapy [9]. Improved proliferation would also decrease the amount of time to therapy for autologous intervention. Across all groups, as much as 2% of the CPCs were visibly dividing (Figure 3(c)). Dividing cells were identified by nuclear condensation, division, and rounded cytoplasm (Figure 3(a)). CPC division was confirmed by the presence of aurora B kinase (Figure 3(b)). A 270-fold difference was observed in CPC proliferation across these culture conditions. The highest observed number of dividing cells was on FN at 5% strain (2.7% dividing) and the lowest was on PLL at 0% strain (0.01% dividing). Unstrained CPCs seeded on FN, COL, or cECM had an increased number of dividing cells (1.2%, 1.0%, and 0.9%, resp.) as compared to CPCs cultured on PLL (*p* < 0.05). At 5% strain, significantly more CPCs were dividing on FN (2.7%) as compared to those on PLL (0.3%; *p* < 0.01) and LN (0.9%; *p* < 0.05). Fibronectin has been demonstrated to be essential for CPC proliferation and survival [35]. The effects of matrix coating are attenuated at 10 and 15% mechanical strains. The proliferative capacity of CPCs is likely underestimated by this method since it only captures dividing cells at a single time point.

**Figure 4:**
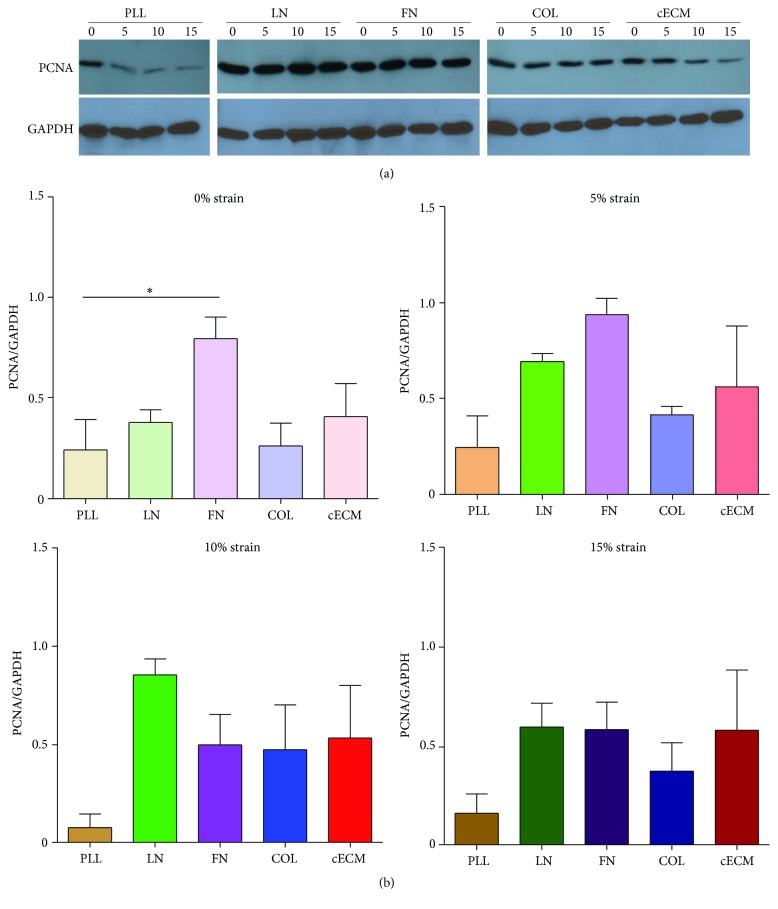
FN induces PCNA expression. CPCs were seeded on each matrix for 6 hours followed by 24 hours of cyclic strain and lysed. Cell lysate was evaluated by western blot. (a) Representative blots. (b) Quantification by densitometry; *n* = 4-5; PLL (brown): poly-L-lysine, LN (green): laminin, FN (purple): fibronectin, COL (blue): collagen I, cECM (red): naturally derived cardiac extracellular matrix, PCNA: proliferating cell nuclear antigen, and *∗* represents *p* < 0.05 by ANOVA.

To address this shortcoming, CPC proliferation was also assessed by expression of proliferating cell nuclear antigen (PCNA). FN induces the highest PCNA expression under static culture conditions (Figure 4). At 5% strain, FN and laminin trended toward increased proliferation. Matrix did not affect the number of dividing cells at 15%, suggesting that mechanical inputs may override the effect of the matrix at higher strains. The upregulation of PCNA by matrix conditions was varied depending on the magnitude of cyclic strain. This suggests that the extracellular matrix surrounding a CPC may dictate the transmission of forces applied to the cell. Two key possibilities may explain this effect. First, the adhesion strength of CPCs may vary by extracellular matrix coating leading to varied levels of basal stress in the cell. Stress across the plasma membrane or cytoskeleton can lead to activation of molecular pathways or changes in chromatin conformation. Alternatively, as biochemical entities, different extracellular matrix proteins may activate different biochemical signaling pathways through adhesion to different cell surface receptors. For example, CPCs may adhere less tightly to LN and therefore require a greater force to induce the same stress in the plasma membrane. This would make them more sensitive to changes in strain magnitude. Future work could investigate these potential mechanisms. Together, the PCNA expression, aurora B kinase staining, and CPC phenotype suggest that CPCs are more likely to proliferate under culture conditions that mimic a niche or infarct microenvironment (i.e., low strain, high FN, and/or LN) than a healthy microenvironment [17]. Culture of CPCs on FN at 5% strain will improve CPC number* in vitro*. This also mimics observations that cells are more proliferative in the young heart, where FN content is high, as compared to the correlation of laminin prevalence in the adult heart with lower proliferative rates [36].

### 3.4. Connexin43 Expression Decreases at Higher Strain Magnitudes

**Figure 5:**
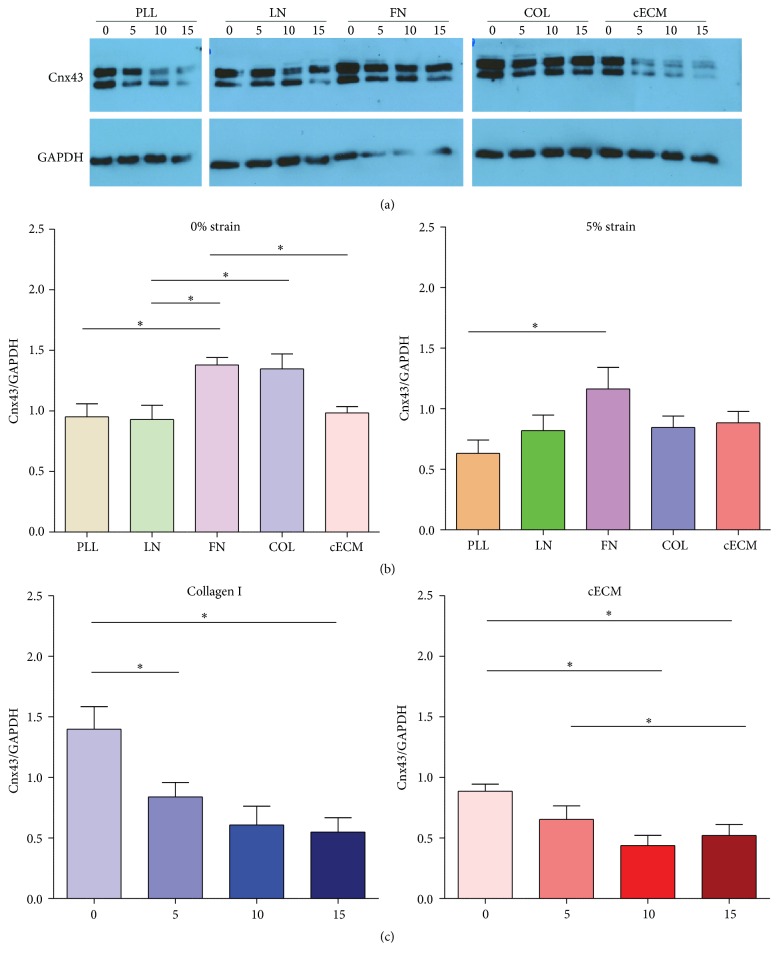
CPC connexin43 expression is highest at low strain magnitudes. CPCs were seeded on each matrix for 6 hours and then cyclic strain was applied for 24 hours. Connexin43 expression was assessed by western blot. (a) Representative blots and (b-c) densitometry quantification. One-way ANOVA with Tukey's multiple comparison test; bars represent mean + SEM; ^*∗*^ *p* < 0.05; *n* = 4–7. (b) Matrix-dependent effects. (c) Strain magnitude-dependent effects. PLL (brown): poly-L-lysine, LN (green): laminin, FN (purple): fibronectin, COL (blue): collagen I, cECM (red): naturally derived cardiac extracellular matrix, 0–15: strain magnitude (%), and Cnx43: connexin43.

Connexin43 (Cnx43) expression facilitates gap junction formation and is important for cardiomyocyte maturation and tissue integration. Alignment and mechanical strain induce Cnx43 expression in relevant cell types [37–39]. Cnx43 expressing cells better integrate when applied to the myocardium [40]. Cnx43 also improves cell survival and proliferation [41, 42]. Western blots were performed to assess Cnx43 expression in strained CPCs. Representative blots and quantification are reported in Figure 5. Unstrained CPCs cultured on FN had 40–50% more Cnx43 (1.4-fold over glyceraldehyde-3-phosphate dehydrogenase) than those cultured on PLL (1.0-fold; *p* < 0.05), LN (0.9-fold; *p* < 0.05), and cECM (1.0-fold; *p* < 0.05). At 5% strain, Cnx43 was maintained on FN (1.2-fold) as compared to that on PLL (0.6-fold; *p* < 0.05). At 10 and 15% strains, Cnx43 expression was not matrix dependent. This suggests that FN is an important signal for Cnx43 expression and that CPCs are more likely to form gap junctions when cultured on FN at low strain magnitudes. Fibronectin also induces Cnx43 expression in neonatal rat ventricular myocytes [32]. However, Cnx43 expression decreased with increasing strain for CPCs on COL (*p* < 0.01) and cECM (*p* < 0.05). This also suggests that CPCs are more likely to form gap junctions under low strain magnitudes. While expression alone is not proof of functional gap junction formation, increased Cnx43 expression suggests that the cells are better primed for functional gap junction formation and electrical propagation. Again, culture of CPCs on FN at low strain magnitudes is the optimal condition for CPC culture.

### 3.5. Strain Induces CPC Alignment

**Figure 6:**
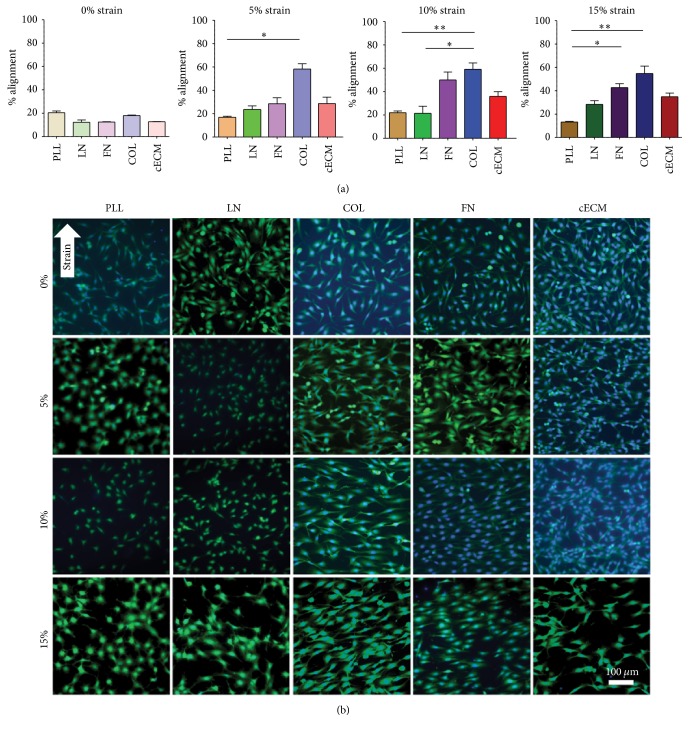
Strain induces CPC alignment. CPCs were seeded on each matrix for 6 hours and then cyclic strain was applied for 24 hours. (a) Quantification of strain-induced matrix-dependent alignment. Kruskal-Wallis one-way ANOVA with Dunn's multiple comparison tests; bars represent mean + SEM; ^*∗*^ *p* < 0.05 and ^*∗∗*^ *p* < 0.01; *n* = 4–7; PLL (brown): poly-L-lysine, LN (green): laminin, FN (purple): fibronectin, COL (blue): collagen I, and cECM (red): naturally derived cardiac extracellular matrix. (b) Representative cell strain images. Blue: DAPI and green: FITC-maleimide; arrow indicates principle direction of strain; 0–15: strain magnitude (%).

Alignment organizes cells to be more tissue-like and has been shown to improve stem cell differentiation [37]. Representative images of CPCs from culture condition are shown in Figure 6(b) and a quantified alignment score is present in Figure 6(a). Consistent with literature, CPCs align perpendicular to the principle axis of applied strain [11]. With the application of 5% strain, more CPCs aligned on COL (58%) than on PLL (18%; *p* < 0.01). At 15%, CPCs aligned better on FN (42%) and COL (54%) as compared to those on PLL (15%; *p* < 0.001). This is expected as cells adhere to PLL through electrostatics rather than integrin binding.

Within each matrix group, the application of strain had an effect on alignment. Notably, CPCs cultured on FN aligned better when strained at 10% (51%; *p* < 0.01) and 15% (42%; *p* < 0.05) strain magnitudes as compared to unstrained (12% alignment) cells. For CPCs cultured on COL, alignment was increased at 5% (58%; *p* < 0.05) and 10% (60%; *p* < 0.05) strain magnitudes as compared to unstrained (17% alignment) cells. Similarly, on cECM there was an increase in alignment at 10 (36%; *p* < 0.05) and 15% (35%; *p* < 0.01) strain magnitudes as compared to unstrained (12% alignment) CPCs. Taken together, CPCs cultured on cECM, FN, or COL had 3- to 4-fold increases in alignment with mechanical strain. This demonstrates that CPCs align with mechanical strain, but that alignment values did not differ within a matrix group with increasing magnitudes of strain above 5%. This suggests that given the appropriate matrix cues and any magnitude of cyclic strain CPCs will align. Therefore, the decrease in Cnx43 and proliferation that is observed with an increase in strain are not due to differences in cell alignment. Future work would benefit from evaluating the differentiation of CPCs under each of these culture conditions.

## 4. Conclusion

It is of great interest to elucidate the interaction of CPCs with their microenvironment to improve engineered microenvironment and cell therapy. This study evaluates two important signaling moieties from the myocardium, extracellular matrix, and mechanical strain, in a combinatorial approach. The regenerative potential of CPCs is assessed through growth factor secretion, proliferation, Cnx43 expression, and CPC alignment. CPCs cultured on FN, COL, and cECM secreted VEGF independent of mechanical strain magnitude. Mechanical strain at a magnitude of 5% improved CPC proliferation and Cnx43 expression. These effects were most profound when the CPCs were cultured on FN. Mechanical strain of all evaluated magnitudes induced alignment of CPCs cultured on FN, COL, and cECM. CPCs have the highest regenerative potential* in vitro* under culture conditions of FN at 5%. These findings highlight the importance of culture condition for CPC behavior. Future work can build upon the importance of FN and low strain magnitude discussed here toward engineering a more complex microenvironment integrating additional signaling cues.

## Abbreviations

CPC: Cardiac progenitor cell
PLL: Poly-L-lysine
LN: Laminin
FN: Fibronectin
COL: Collagen I
cECM: Naturally derived cardiac extracellular matrix
PCNA: Proliferating cell nuclear antigen
Cnx43: Connexin43
VEGF: Vascular endothelial growth factor.
